# Resting Body Temperature and Long-Term Survival in Older Adults at a Mental Health Center: Cross-Sectional and Longitudinal Data

**DOI:** 10.3390/jcm14030713

**Published:** 2025-01-22

**Authors:** Piotr Paweł Chmielewski, Bartłomiej Strzelec, Krzysztof Data, Krzysztof Chmielowiec, Paul Mozdziak, Bartosz Kempisty

**Affiliations:** 1 Division of Anatomy, Department of Human Morphology and Embryology, Faculty of Medicine, Wroclaw Medical University, 6a Chalubinskiego Street, 50-368 Wrocław, Poland; 2 2nd Department of General Surgery and Surgical Oncology, Medical University Hospital, 50-556 Wroclaw, Poland; 3 Department of Hygiene and Epidemiology, Collegium Medicum, University of Zielona Góra, 65-046 Zielona Góra, Poland; 4 Prestige Department of Poultry Science, College of Agriculture and Life Sciences, North Carolina State University, Raleigh, NC 27695-7608, USA; 5 Department of Veterinary Surgery, Institute of Veterinary Medicine, Nicolaus Copernicus University, 87-100 Toruń, Poland; 6 Center of Assisted Reproduction, Department of Obstetrics and Gynecology, University Hospital and Masaryk University, 625 00 Brno, Czech Republic

**Keywords:** body temperature, biomarkers, health outcomes, longevity, survival, thermoregulation

## Abstract

**Background/Objectives:** Elevated body temperature is a well-established biomarker of infection, increased disease risk, and adverse health outcomes. However, the relationship between resting body temperature and long-term survival in older individuals is complex. Emerging evidence suggests that higher basal body temperature is associated with reduced survival and accelerated aging in non-obese older adults. This study aimed to compare body temperatures across different age groups in hospitalized older adults. **Methods:** Data were retrospectively collected from 367 physically healthy residents of a mental health center. Longitudinal data from 142 individuals (68 men and 74 women), aged 45 to 70 years and monitored continuously over 25 years, were compared with cross-sectional data from 225 individuals (113 men and 112 women) who underwent periodic clinical examinations with temperature measurements. The cross-sectional sample was stratified into four survival categories. Resting oral temperatures were measured under clinical conditions to ensure protocol consistency. Age-related changes in both sexes were evaluated using standard regression analysis, Student’s *t*-tests, ANOVA, and Generalized Linear Models. **Results:** Longitudinal analysis revealed an increase in body temperature with age among women, while cross-sectional analysis showed that long-lived residents generally had lower body temperatures compared to their shorter-lived counterparts. **Conclusions:** These findings support the hypothesis that lower lifetime steady-state body temperature is associated with greater longevity in physically healthy older adults. However, further research is needed to determine whether the lower body temperature observed in long-lived individuals is linked to specific health advantages, such as enhanced immune function, absence of detrimental factors or diseases, or a reduced metabolic rate potentially influenced by caloric restriction.

## 1. Introduction

Identifying reliable predictors of long-term survival remains a critical challenge in both medicine and aging research [1,2,3,4]. Body temperature, which reflects the balance between heat production and dissipation, is a fundamental indicator of general health [5], although its role in predicting survival requires further exploration. Research suggests that lower basal body temperature is a biomarker of healthy aging and longevity in older adults [6,7]. However, it remains unclear whether lower body temperature directly contributes to increased longevity or serves as a proxy for better health, such as more effective immune responses or the absence of diseases [8,9]. Unhealthy lifestyle factors, including high-calorie diets, chronic stress, and sleep deprivation, as well as undiagnosed conditions like type 2 diabetes and latent infections, have been linked to elevated body temperature and reduced survival [6,10]. In contrast, conditions such as advanced sarcopenia, atherosclerosis, and other circulatory disorders, which lower body temperature, are strongly associated with increased mortality in older adults.

The hypothalamus tightly regulates core body temperature to maintain homeostasis. Neurons in the preoptic area integrate central and peripheral signals to keep core temperature stable by modulating autonomic and hormonal control of heat production and loss [9]. Maintaining a body temperature higher than the ambient temperature requires significant energy expenditure. When food is scarce, reducing core body temperature conserves energy, which is a strategy linked to the metabolic and molecular adaptations of caloric restriction (CR). In healthy adults, body temperature follows a circadian rhythm, generally lower in the morning and higher in the afternoon [11,12]. This pattern reflects a dynamic balance between heat generated by metabolism and heat lost to the environment. However, temperature readings taken at arbitrary times may not accurately represent the body’s temperature set point because of daily fluctuations and external influences [7,12]. Fever is an adaptive defense mechanism that protects the body against pathogens, playing a crucial role in the immune response [13,14]. Fever is induced by pyrogens, which can be exogenous (e.g., bacterial lipopolysaccharides acting on Toll-like receptors) or endogenous (e.g., proinflammatory cytokines such as IL-1, IL-6, TNF-β, and IFN-γ) [15,16].

Mean body temperature decreases with advancing age, as older adults exhibit impaired thermoregulation due to reduced metabolic rate, sarcopenia, and vascular changes [17,18,19,20]. These factors diminish their ability to generate and regulate heat effectively. Despite the availability of established predictors of survival, including clinical markers (e.g., blood pressure, cholesterol levels, etc.), diagnostic indicators (e.g., imaging results), and health-related behaviors (e.g., exercise, smoking, alcohol use, participation in health screening examinations, etc.), these metrics often lack the integrative nature of basal body temperature, which reflects the dynamic interplay of multiple physiological systems. Unlike these individual metrics, basal body temperature serves as a cumulative indicator of metabolic efficiency, inflammatory status, and thermoregulatory capacity in the course of an individual’s aging process. In spite of its potential relevance, the role of body temperature as a biomarker of longevity remains understudied, largely due to the scarcity of longitudinal research examining its relationship with long-term survival in physically healthy older adults. Most existing data come from ill or hospitalized patients, limiting insights into how temperature varies with age in physically healthy individuals.

The current study addresses this by employing cross-sectional and longitudinal data to test the hypothesis that lower steady-state body temperature is associated with greater longevity in physically healthy, non-obese older adults. This research aims to advance understanding of the relationship between resting body temperature and long-term survival, contributing to the medical literature and informing future studies, such as meta-analyses.

## 2. Materials and Methods

### 2.1. Study Design and Participants

This study complied with the principles of the Declaration of Helsinki and utilized archival data from physical examinations conducted at the Mental Health Center near Zielona Góra, Lubuskie Province, Poland. Approved by the relevant ethical authorities in 2007 as part of a PhD research project, the study adhered to all applicable legal and ethical standards. To ensure patient confidentiality, all medical records were anonymized and used to construct a database incorporating both longitudinal and cross-sectional data.

The longitudinal cohort, consisting of 68 men and 74 women, was continuously monitored from ages 45 to 70 years. In contrast, the cross-sectional cohort, comprising 113 men and 112 women, was assessed during periodic clinical examinations conducted at multiple intervals for each individual. Across both cohorts, participants’ health status was regularly monitored through routine clinical examinations under the same conditions at the same medical institution throughout the study period.

The cross-sectional cohort was stratified into four lifespan categories based on death certificates: (1) short (22 men, aged 50–58 years, mean age 53 years; 12 women, aged 50–58 years, mean age 53 years), (2) medium (27 men, aged 58–65 years, mean age 63 years; 30 women, aged 58–65 years, mean age 63 years), (3) long (49 men, aged 65–72 years, mean age 68 years; 40 women, aged 65–72 years, mean age 68 years), and (4) very long (15 men and 30 women, all aged 76 years and above).

It is important to note that the first category included only individuals with short lifespans (<*e*_0_, life expectancy at birth), the next two categories consisted of individuals with medium and long lifespans, respectively, while the oldest category comprised only long-lived individuals, all of whom had reached or surpassed the age of 76 years, thus exceeding the value of *e*_0_.

### 2.2. Measurements

All measurements were conducted systematically by trained medical personnel under standardized clinical conditions at a consistent time of day, typically in the morning. Analyses were performed in the hospital laboratory using uniform methods, ensuring consistency across participants. Resting body temperature (°C) was measured sublingually each month using a calibrated thermometer with an accuracy of 0.1 °C. Body height was recorded to the nearest 0.1 cm with participants standing in standardized posture, while weight was measured to the nearest 0.1 kg using calibrated medical scales. Blood pressure was assessed after a brief rest using a Riva-Rocci sphygmomanometer, whereas heart rate was determined by palpating the carotid artery. Hematological data were extracted from medical records.

All calculations and statistical analyses in this study were based on aggregated data derived from repeated measurements per participant over the study period, encompassing approximately 300 measurements per individual in the longitudinal dataset and at least 36 measurements per individual in the cross-sectional dataset. Even the smallest subgroup, consisting of 12 women, contributed substantial observational data (12 individuals × 36 measurements = 432 observations), ensuring a robust foundation for analysis. For each participant, repeated measurements were averaged to provide reliable estimates of central tendency and variability, including means, standard deviations (SDs), standard errors, confidence intervals, and so forth, which were subsequently used in the final analyses. This methodological approach enhanced statistical rigor and precision, minimizing variability and ensuring the reliability and representativeness of the findings.

### 2.3. Statistical Methods

To assess age-related changes in body temperature in both sexes, *t*-tests and standard regression analyses were conducted. The method of least squares was employed. Five types of regression functions were tested to identify the best-fit model: (1) linear function, y = *β*_1_ × age + *β*_0_; (2) logarithmic function, y = β_1_ × age + *β*_0_; *β*_1_ ln(age) + *β*_0_; (3) polynomial function, y = *β*_1_ × age_2_ + *β*_2_ × age + *β*_0_; (4) exponential function type I, y = *β*_1_ × age a; and (5) exponential function type II, y = *β*_1_ × ea × age, where x represents age (the independent variable), y stands for the value of the characteristic analyzed during the aging process, *β*_2_ denotes the second regression coefficient, a is the exponent, and *e* is the base of the natural logarithm. The goodness of fit for a given model was confirmed when the coefficient of determination (*R*^2^) reached its highest value and both the intercept (*β*_0_) and the regression coefficient (*β*_1_) were statistically significant (*p* < 0.05).

To validate the robustness of the results, sensitivity analyses were conducted using alternative statistical models and subsampling approaches. These analyses produced consistent findings, reinforcing the reliability of the observed trends. In addition to the primary calculations and statistical analyses, which included Student’s *t*-tests and standard regression analyses, ANOVA, Generalized Linear Models (GLM), and post-hoc least-significant-difference (LSD) tests were employed to assess the effects of age, sex, and their interaction on body temperature changes. GLM analyses were applied to both longitudinal and cross-sectional datasets.

Power analyses, based on anticipated effect sizes and a significance level of 0.05, confirmed that the longitudinal cohort of 142 individuals and the cross-sectional cohort of 225 individuals provided sufficient statistical power (>0.8) to detect age-related changes. These calculations underscore the robustness and representativeness of the datasets. All analyses were performed using the Statistica package version 13.1 (StatSoft, Inc, Tulsa, OK, USA). 

## 3. Results

**Figure 1:**
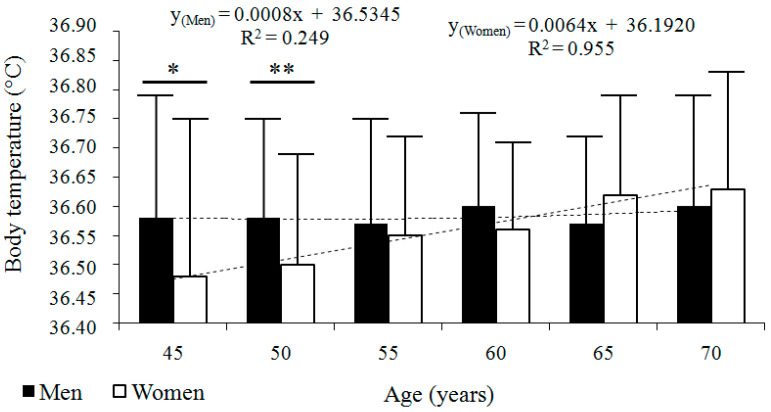
Longitudinal changes in body temperature (°C) across six consecutive age categories in men (*n* = 68) and women (*n* = 74). Data are presented as arithmetic means ± standard deviations (SD), accompanied by regression models and their corresponding coefficients of determination (*R*^2^). * *p* ≤ 0.05, ** *p* ≤ 0.01, differences between sexes compared with Student’s *t*-test.

The baseline characteristics of the longitudinal sample are presented in Table 1. In the two samples, body temperature was normally distributed (K–S test, *p* value > 0.2). The longitudinal analysis revealed that body temperature fluctuated throughout the study period. No significant age-related trend was observed in men. However, in women, body temperature increased across the six consecutive age categories. The best-fitted regression model was linear in both sexes: for men, y = 0.0008x + 36.5345; *R*^2^ = 0.249; and for women, y = 0.0064x + 36.192; *R*^2^ = 0.955 (Figure 1). Men and women did not differ significantly in mean body temperature, except in the first two age categories, when men had higher body temperatures than women (Table 1 and Table 2).

**Figure 2:**
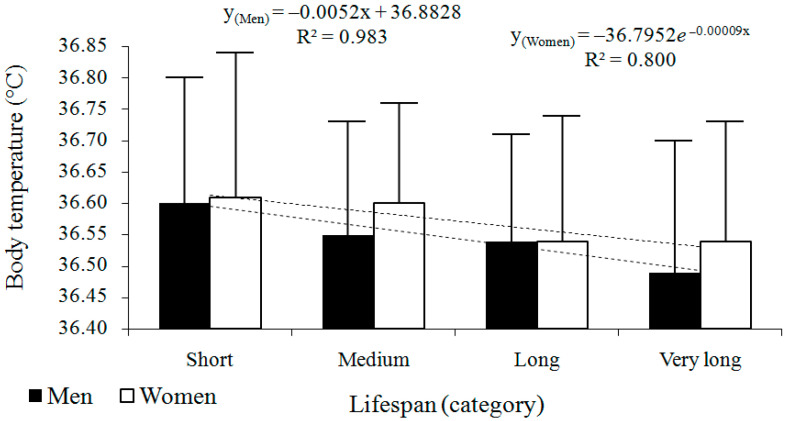
Cross-sectional changes in body temperature (°C) across four survival categories in men (*n* = 113) and women (*n* = 112). Data are presented as arithmetic means ± standard deviations (SD), accompanied by regression models and their corresponding coefficients of determination (*R*^2^).

The cross-sectional analysis showed that body temperature decreased with advancing age in both sexes (Figure 2, Table 3). Lower age at death was generally associated with higher body temperature, while higher at death corresponded to lower body temperature. The best-fitted regression model was linear in men (*y* = −0.0052x + 36.8828; *R*^2^ = 0.983) and exponential in women (*y* = −36.7952*e*^−0.00009x^; *R*^2^ = 0.8).

**Figure 3:**
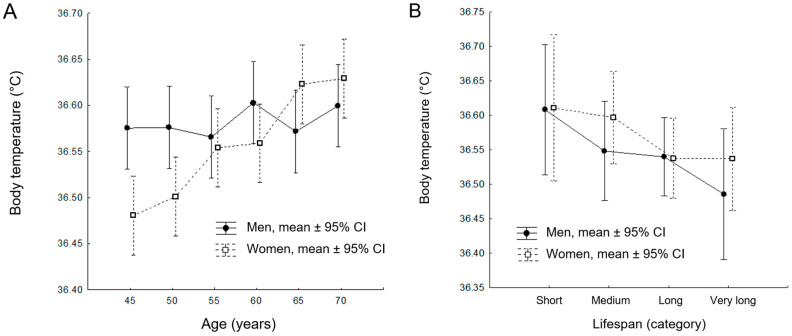
Results of the Generalized Linear Model (GLM) analysis for the longitudinal cohort (**A**) and cross-sectional cohort (**B**). Mean values and confidence intervals (CI) are presented based on robust statistical calculations and analyses.

The results of the GLM analysis for the longitudinal dataset revealed a statistically significant model of age-related changes in body temperature (*R*^2^ = 0.051; *F*_11,840_ = 4.089; *p* < 0.001; Figure 3A). Specifically, age had a significant effect on body temperature changes (*F*_5,840_ = 4.598; *p* < 0.001; *η*^2^ = 0.027; Table 4), as did the interaction between age and sex (*F*_5,840_ = 3.381; *p* = 0.005; *η*^2^ = 0.020; Table 4). These findings further support the primary observation that body temperature is affected by aging.

However, sex alone did not have a statistically significant effect on body temperature changes in the longitudinal sample (*F*_5,840_ = 3.523; *p* = 0.061; *η*^2^ = 0.004). The results of the *post-hoc* LSD test for the longitudinal dataset are detailed in Table 5.

For the cross-sectional sample, the GLM procedure did not reveal a statistically significant model *R*^2^ = 0.033; *F*_7,196_ = 0.968; *p* = 0.455; Figure 3B). Neither age at death, sex, nor the interaction between age and sex had a significant effect on body temperature in the cross-sectional sample (*F*_3,196_ = 0.307; *p* = 0.820; Table 6). The results of the post-hoc LSD test for the cross-sectional dataset are detailed in Table 7.

## 4. Discussion

This study examined the relationship between resting body temperature and long-term survival in two cohorts of physically healthy older adults. In the longitudinal cohort, we observed an age-related increase in body temperature among women, while no such trend was evident in men. Conversely, the cross-sectional analysis indicated that individuals with shorter lifespans tended to have higher resting body temperatures, whereas those with longer lifespans generally had lower temperatures. These findings suggest a potential link between lower resting body temperature and greater longevity in this population. Participants were selected based on the availability of comprehensive archival records, encompassing a broad demographic within the region. The uniformity of their living conditions and baseline physical health (Table 1), minimizes environmental variance, enhancing the reliability of our findings. Moreover, our results are consistent with prior studies, further supporting their generalizability.

The robustness of our findings was confirmed through GLM analyses, which demonstrated significant effects of age and age–sex interactions on resting body temperature in the longitudinal cohort. This underscores the unique strength of longitudinal designs in detecting dynamic, age-related changes in physiological parameters, as these changes may remain undetectable in cross-sectional analyses. Indeed, the absence of statistically significant results in the cross-sectional sample, despite trends observed in the longitudinal data, highlights the inherent limitations of cross-sectional approaches, which often lack the sensitivity to capture cumulative or nuanced physiological changes over time.

Several potential mechanisms may explain these findings. For example, chronic low-grade systemic inflammation (CLSI, also referred to as inflammaging), which has recently been recognized as one of the hallmarks of aging, can contribute to elevated body temperature and reduced longevity [21,22]. Furthermore, age-related conditions such as insulin resistance, type 2 diabetes, and autoimmune diseases, all of which drive CLSI, might also be involved in comorbidities [23]. In general, women are more susceptible to autoimmune disorders than men [24,25]. In this study, women had significantly higher body mass index (BMI) than men [26]. It is well-established that high BMI is linked not only to increased mortality but also to elevated white blood cell (WBC) counts, both of which correlate with reduced longevity [27,28].

**Figure 4:**
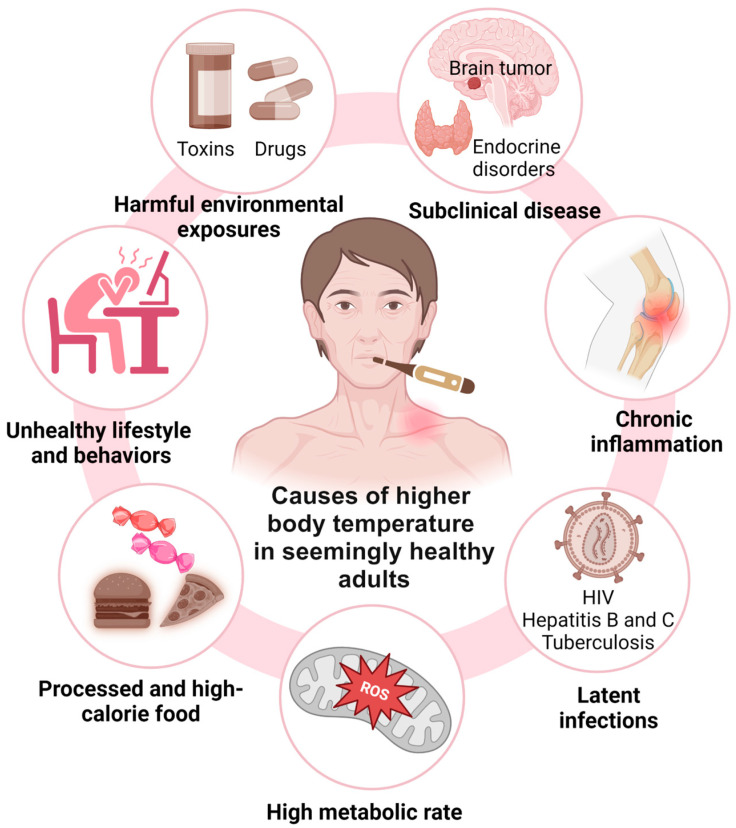
Seven groups of factors that might contribute to the association between higher body temperature and reduced long-term survival in seemingly healthy older adults. These factors include subclinical disease, systemic inflammation, latent infections, high metabolic rates, unhealthy lifestyle and behaviors, including consumption of processed and high-calorie foods, and harmful environmental exposures (see text for detail).

Research indicates that a lower core body temperature might serve as a biomarker of longevity, influenced by various factors affecting both thermoregulation and survival (Figure 4). These factors include the following: (1) the absence of subclinical conditions, such as brain tumors or hyperthyroidism, (2) a reduced risk of CLSI associated with conditions like insulin resistance, type 2 diabetes mellitus, and autoimmune disorders, which can impair health and longevity [23], (3) the absence of latent infections such as tuberculosis, human immunodeficiency viruses (HIV), and hepatitis B and C, which can elevate body temperature and increase the risk of premature death [29,30,31], (4) a lower metabolic rate, which is linked to reduced molecular and cellular damage due to decreased reactive oxygen species (ROS) production—a benefit potentially enhanced by limited caloric intake, which is also associated with lower body temperature and may promote longevity [8,32], (5) the avoidance of unhealthy lifestyle factors, including chronic psychological stress, sleep deprivation, physical inactivity (sedentarism), high-calorie diets, and increased body fatness (e.g., obesity), which can raise the body’s core temperature and concurrently reduce long-term survival [33,34,35,36], and (6) minimizing prolonged exposure to harmful environmental factors such as high ambient temperatures or toxins [37,38,39].

**Figure 5:**
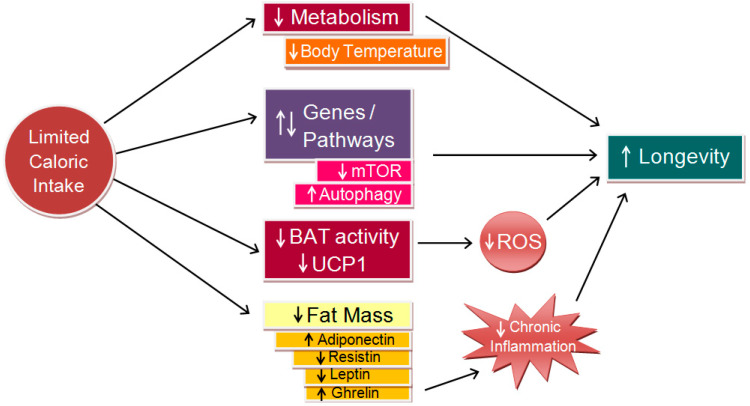
Conceptual model linking caloric restriction (CR) to longevity (adapted from [8], modified). CR lowers metabolic rate, which in turn decreases body temperature and mitigates molecular and cellular damage. This reduction curtails the production of reactive oxygen species (ROS), thereby decreasing oxidative stress. Collectively, these effects can promote health and longevity through mechanisms such as mTOR inhibition and autophagy enhancement. Additionally, they alleviate chronic systemic inflammation, further contributing to increased longevity. BAT = brown adipose tissue; UCP1 = uncoupling protein one.

Carrillo and Flouris (2011) proposed a model linking CR, body temperature, and longevity, supported by robust evidence showing that CR delays aging and extends lifespans across various species (Figure 5) [8]. CR induces a chronic negative energy balance, reducing fat mass and altering adipokine levels, such as adiponectin, resistin, and leptin, thus stimulating appetite via ghrelin secretion. These changes are linked to lower levels of circulating proinflammatory cytokines, partly due to ghrelin’s inhibition of leptin-induced cytokine expression [40]. In white adipose tissue, CR up-regulates genes involved in glucose, amino acid, and fatty acid metabolism, as well as mitochondrial energy processes, enhancing fuel efficiency and uptake [41]. Concurrently, CR down-regulates inflammation-related genes, including those encoding cytokines and acute phase proteins [42]. While the relationships between CR, reduced fat mass, and lower body temperature are well-established, the putative influence of lower body temperature on gene expression requires further investigation.

Soare et al. (2011) found that long-term CR, but not endurance exercise, consistently lowers core body temperature in weight-stable and lean individuals [9]. This reduction in 24 h core body temperature among CR practitioners likely reflects a protective physiological adaptation geared towards conserving energy. This interpretation is bolstered by the observation that lower core body temperature in the CR group is accompanied by reduced circulating levels of triiodothyronine, insulin, leptin, and testosterone—hormones crucial for nutrient sensing and metabolic regulation [43,44]. The simultaneous decline in core body temperature and these anabolic hormones suggests a physiological state finely attuned to energy restriction. Moreover, these findings indicate that long-term CR, when adequate nutrition is maintained, can lead to a reduction in body temperature across 24 h, daytime, and nighttime periods in healthy individuals, thus paralleling the effects observed in animal models such as rodents and primates.

However, in the Baltimore Longitudinal Study of Aging (BLSA), men with a core body temperature below the median lived significantly longer than those with a body temperature above the median, even in the absence of CR, suggesting that lower body temperature may independently contribute to enhanced longevity [45]. These findings are in line with the theory of molecular entropy, which posits that biological aging is driven by the accumulation of molecular damage and increasing disorder within biological systems, which results in a gradual loss of functional integrity over time [46,47]. This process is influenced by both genetic and environmental factors, including metabolic activity, which generates ROS and other byproducts that contribute to molecular damage.

Furthermore, it can be argued that a higher body temperature reflects increased kinetic energy within the body’s cells, which in turn could elevate metabolic activity. This heightened metabolic rate is associated with greater production of ROS and other free radicals, which are known to cause oxidative damage to cellular components, including DNA [48,49,50,51,52,53,54,55,56]. Such damage accelerates the shortening of telomeres, i.e., protective caps on chromosomes, whose attrition triggers cellular senescence—a fundamental process that contributes to aging and limits the length of life [50,57,58,59]. On the other hand, several studies have reported that moderate increases in temperature (e.g., through exercise or thermal baths) can have beneficial effects on health, possibly due to hormesis, i.e., a process where low levels of stress stimulate adaptive responses that enhance longevity [60,61,62,63].

Lower core body temperature may reduce metabolic rate, subsequently decreasing the production of ROS and other entropy-generating byproducts. Thus, a reduced metabolic rate, which is associated with lower core body temperature, might slow down the rate of molecular entropy accumulation, thereby preserving cellular function and integrity for a longer period [46,47]. This hypothesis suggests that lower core body temperature could independently mitigate the entropic forces of aging [46], decelerating the deterioration of cellular structures and functions. This effect may operate in parallel with, or even independently of, other longevity-promoting interventions like CR [47]. Consequently, individuals with naturally lower core body temperatures might experience a slower progression of aging due to a more stable and orderly molecular environment, possibly leading to extended lifespan.

Waalen and Buxbaum (2011) reported higher body temperatures in women and a significant decline in body temperature with advancing age in both sexes [6]. Their observation that obesity was linked to elevated WBC counts and higher body temperatures supported the hypothesis that lower resting body temperature could be a biomarker of longevity in physically healthy older adults. These authors concluded that after controlling for sex, BMI, and WBC count, mean body temperature decreased with age, with a difference of 0.3° F between oldest and youngest groups.

Finally, Simonsick et al. (2016) demonstrated that lower basal body temperature correlates with indicators of healthy aging, such as faster gait speed, better endurance, and lower perceived exertion during walking, particularly in non-adipose older adults [7]. These associations remained robust even after controlling for factors such as age and sex. However, in individuals with excessive adiposity, these relationships were reversed, with lower body temperature linked to slower gait and diminished walking performance over time. Furthermore, no significant associations were found between basal temperature and grip strength.

Overall, while lower basal body temperature is associated with markers of healthy aging in non-obese and healthy older adults, its reliability as a genuine biomarker of healthy aging diminishes in the presence of excessive adiposity or declining health. This underscores the importance of considering individual health status when evaluating biomarkers of healthy aging and longevity, as the relationship between basal body temperature and long-term survival may be compromised by ill health, including frailty and other geriatric syndromes [64,65,66,67,68,69,70,71,72,73,74,75].

It is important to acknowledge certain limitations of this study. The relatively specific and modest sample size, along with the clinical setting in which all measurements were conducted, may limit the generalizability of the findings to broader populations. Future research should aim to replicate and expand upon these results by including more diverse cohorts and exploring a wider spectrum of physiological parameters. Such efforts would deepen our understanding of the intricate relationship between resting body temperature and longevity in physically healthy older adults.

## 5. Conclusions

This study sheds further light on the complex relationship between resting body temperature and longevity in physically healthy older adults. Cross-sectional data indicate that individuals with lower body temperatures tend to live longer, supporting the hypothesis that a lower steady-state body temperature is associated with greater longevity. Although these findings highlight the potential of body temperature as a biomarker of health aging and longevity, further research is required to determine whether this association is mediated by other factors such as metabolic rate, immune function, or restricted caloric intake.

## Figures and Tables

**Table 1:** Baseline characteristics of the study individuals at the age of 45 years. Statistically significant differences (*p*-values) are in bold.

Variable	Men (*n* = 68)		Women (*n* = 74)		*t*-Test	*p*-Value
	Mean	SD	Mean	SD		
**Body height, cm**	**169.7**	6.7	157.1	7.2	11.1	**<0.001**
Body weight, kg	66.4	8.9	61.7	11.6	2.7	**0.009**
BMI	23.0	2.7	25.0	4.4	−3.3	**0.001**
HR, bpm	82.7	8.1	82.1	9.2	0.4	0.718
Systolic BP, mmHg	121.2	10.8	119.8	12.3	0.7	0.470
Diastolic BP, mmHg	76.3	6.8	75.8	8.0	0.4	0.702
Leukocyte count, 10^3^/μL	6.8	1.5	6.3	2.0	1.5	0.135
Monocytes, %	2.0	1.7	2.3	2.4	0.7	0.500
Granulocytes, %	68.0	8.1	66.5	7.0	1.2	0.228
Eosinophils, %	3.3	2.5	3.3	2.5	0.1	0.953
Lymphocytes, %	30.6	8.1	31.8	6.8	1.0	0.317
NLR	2.3	1.2	2.0	0.7	1.6	0.110
Body temperature, °C	36.6	0.2	36.5	0.3	2.3	**0.024**

BMI = body mass index, BP = blood pressure, HR = heart rate, NLR = neutrophil-to-lymphocyte ratio. Values are arithmetic means ± standard deviations (SD).

**Table 2:** Longitudinal changes with age in body temperature (°C) in men and women (*n* = 142) aged 45 through 70 years in the six consecutive age categories. Statistically significant differences (*p*-values) are in bold.

Age	Men (*n* = 68)		Women (*n* = 74)		*t*-Test	*p*-Value
	Mean	SD	Mean	SD		
45	36.58	0.21	36.48	0.27	2.29	**0.024**
50	36.58	0.17	36.50	0.19	2.49	**0.014**
55	36.57	0.18	36.55	0.17	0.40	0.687
60	36.60	0.16	36.56	0.15	1.71	0.090
65	36.57	0.15	36.62	0.17	−1.91	0.058
70	36.60	0.19	36.63	0.20	−0.90	0.367

**Table 3:** Cross-sectional changes with age in body temperature (°C) in men and women (*n* = 225) from four different categories of survival.

Category of Survival	Men (*n* = 113)			Women (*n* = 112)			*t*-Test	*p*-Value
	Mean	SD	*n*	Mean	SD	*n*		
Short	36.61	0.20	22	36.61	0.23	12	0.00	0.975
Medium	36.55	0.18	27	36.60	0.16	30	0.05	0.286
Long	36.54	0.17	49	36.54	0.20	40	0.00	0.961
Very long	36.49	0.21	15	36.54	0.19	30	0.05	0.437

**Table 4:** The GLM results for the longitudinal sample in six consecutive age categories. Statistically significant differences (*p*-values) are in bold.

	*df*	*F*	*p*	*η* ^2^	Power *α* = 0.05
Intercept	1	324 × 10^5^	**<0.001**	0.999	1.000
Age	5	4.598	**<0.001**	0.027	0.975
Sex	1	3.523	0.061	0.004	0.466
Age × Sex	5	3.381	**0.005**	0.020	0.906
Error	840				

**Table 5:** The post-hoc LSD test for the longitudinal sample in six consecutive age categories. Statistically significant differences (*p*-values) are in bold.

Age/Sex	{1} 36.57	{2} 36.48	{3} 36.58	{4} 36.50	{5} 36.57	{6} 36.55	{7} 36.60	{8} 36.56	{9} 36.57	{10} 36.62	{11} 36.60	{12} 36.63
{1} 45/M		**0.003**	0.987	**0.018**	0.763	0.496	0.391	0.598	0.906	0.131	0.455	0.088
{2} 45/F			**0.003**	0.506	**0.007**	**0.017**	**0.000**	**0.011**	**0.004**	**0.000**	**0.000**	**0.000**
{3} 50/M				**0.017**	0.751	0.486	0.400	0.587	0.894	0.135	0.464	0.091
{4} 50/F					**0.040**	0.086	**0.001**	0.061	**0.025**	**0.000**	**0.002**	**0.000**
{5) 55/M						0.709	0.246	0.826	0.854	0.069	0.294	**0.044**
{6} 55/F							0.120	0.875	0.575	**0.025**	0.149	**0.015**
{7} 60/M								0.161	0.329	0.525	0.912	0.406
{8} 60/F									0.684	**0.038**	0.197	**0.023**
{9} 65/M										0.103	0.387	0.068
{10} 65/F											0.454	0.842
{11} 70/M												0.345
{12} 70/F												

**Table 6:** The GLM results for the cross-sectional sample in four categories of survival. Statistically significant differences (*p*-values) are in bold.

	*df*	*F*	*p*	*η* ^2^	Power *α* = 0.05
Intercept	1	651 × 10^4^	**<0.001**	0.999	1.000
Age	3	1.804	0.148	0.027	0.465
Sex	1	0.768	0.382	0.004	0.141
Age × Sex	3	0.307	0.820	0.005	0.109
Error	196				

**Table 7:** The post-hoc LSD test for the cross-sectional sample in four categories of survival.

Age/Sex	{1} 36.61	{2} 36.61	{3} 36.55	{4} 36.60	{5} 36.54	{6} 36.54	{7} 36.49	{8} 36.54
{1} 53/M		0.971	0.320	0.844	0.223	0.213	0.073	0.245
{2} 53/F			0.335	0.823	0.244	0.234	0.084	0.261
{3} 63/M				0.331	0.857	0.826	0.301	0.829
{4} 63/F					0.202	0.192	0.061	0.241
{5} 68/M						0.962	0.334	0.949
{6} 68/F							0.355	0.983
{7} >76/M								0.405
{8} >76/F								

## Data Availability

The data presented in this study are available on request from the corresponding author, as these data are not publicly available due to privacy and ethical restrictions.
